# Fructose Metabolism and Metabolic Dysfunction in Adolescents and Young Adults

**DOI:** 10.3390/nu15143162

**Published:** 2023-07-16

**Authors:** Samir Softic, Miguel A. Lanaspa, Brian DeBosch

**Affiliations:** 1 Division of Gastroenterology, Hepatology, Nutrition, Department of Pediatrics, University of Kentucky College of Medicine and Kentucky Children’s Hospital, Lexington, KY 40536, USA; 2 Department of Pharmacology and Nutritional Sciences, University of Kentucky College of Medicine, Lexington, KY 40506, USA; 3 Division of Endocrinology, Metabolism and Diabetes, Department of Medicine, University of Colorado Anschutz Medical Campus, Aurora, CO 80045, USA; 4 Department of Pediatrics and Cell Biology & Physiology, Washington University School of Medicine, St. Louis, MO 63110, USA

There is a worldwide epidemic of obesity and its associated metabolic dysfunction. Dietary fructose intake is a risk factor for the development of poor metabolic health. In this Special Issue of *Nutrients* titled “Fructose Metabolism and Metabolic Health Effects”, we compiled a series of manuscripts reporting on the detrimental health effects of dietary fructose. The first two manuscripts interrogate fructose intake on human health, one manuscript broadens our mechanistic understanding of fructose-induced metabolic pathways in mice, and the last two manuscripts offer in-depth reviews of fructose intake on the gut microbiome and developmental reprogramming. Together these reports advance our understanding of this complex sugar and bring us closer to fully identifying the metabolic sequelae of excess dietary fructose intake.

Fructose is a monosaccharide most commonly consumed as a caloric sweetener in the form of high-fructose corn syrup (HFCS) or table sugar, sucrose. In this Special Issue, Sigala et al. examined the effects of HFCS comprising 0, 10, 17.5, or 25 percent of the daily energy requirements for two weeks in eighty-five young adults 18–40 years of age [1]. They found a dose-dependent increase in hepatic lipid content and a decrease in insulin sensitivity with increasing doses of dietary fructose intake. This study suggests that even short-term fructose intake in the amounts commonly consumed in our diets is sufficient to support the development of metabolic dysfunction. 

On the other hand, rather than studying added fructose, Radulescu et al. studied the effects of dietary counseling aimed at reducing the intake of obesogenic foods in a real-life clinical setting in one hundred and sixty-five patients aged 2–18 years old [2]. They found that subjects who were able to reduce their body mass index consumed significantly fewer foods that were high in sugar compared to the subjects who were not successful in reducing their BMI. Interestingly, both reports studied fructose intake in predominantly adolescent or young adult populations. Notably, adolescents and young adults are the two age groups that consume more sugar than any other group [3]. Therefore, these groups may be ideal target populations to advocate for reducing their sugar intake. 

There are several proposed mechanisms by which fructose metabolism supports the development of metabolic dysfunction. They include the strong propensity of fructose to induce hepatic de novo lipogenesis [4,5], decrease fatty acid oxidation [6,7], increase uric acid production [8,9], and, more recently, enhance protein acetylation [10], which induces protein misfolding and ER stress [11]. The manuscript by Doridot et al. used a systems approach to identify the pathways in the liver that mediate fructose-induced metabolic dysfunction [12]. They found that the pathways mediating an increase in serum triglycerides and insulin are distinct from those that associate with fructose-mediated changes in body weight and liver triglycerides. They conclude that multiple independent mechanisms may contribute to different aspects of fructose-induced metabolic disease, a concept that unifies the studies mentioned above. 

The numerous deleterious effects on metabolic health are not only confined to the subject consuming high amounts of dietary fructose. Hsu et al. reviewed the current evidence supporting the link between maternal fructose intake and developmental reprogramming in offspring [13]. They focused on gut microbiota and gut microbiota-targeted therapy as a mediator of developmental programming that primes for adult-onset disease or as a potential approach to mitigate the global burden of fructose-related disorders, respectively. In the same light, Thompson et al. extended the review of the evidence linking a maternal fructose diet and offspring microbiome but also summarized the effect on offspring hypertension, glucose tolerance, lipid metabolism, cognition, and retinopathy [14]. These reviews underscore the importance of fructose restriction, not only for the subjects at risk but also for subsequent generations. Moreover, similar to the aforementioned studies in humans, these reviews address young female patients of reproductive age as the target population for intervention. 

In summary, dietary fructose intake is a risk factor for the development of metabolic dysfunction. Even moderate, short-term fructose intake can impair metabolic health, whereas fructose restriction has the potential to improve obesity and its associated complications. There are multiple simultaneous mechanisms by which fructose exerts these functions, which explains the strong penetrance of its effects not only in directly exposed subjects but also in their subsequent generations. The primary target population to benefit from fructose restriction or pharmacotherapy targeting fructose metabolism may be adolescents and young adults, as metabolic dysfunction in this age group is driven by the severity of obesity despite the relatively young age of the patients [15]. Overall, these studies advance our understanding of how excess dietary fructose impacts metabolic health and how specific interventions can mitigate these impacts. Nevertheless, the continued interrogation of the mechanisms that drive and optimal interventions against fructose-induced metabolic disease remains an important avenue through which to combat the ongoing epidemic of obesity and metabolic syndrome. 
